# Welche Migränepatienten werden mit CGRP(R)-Antikörpern behandelt? Ein Vergleich zu unspezifischen oralen Prophylaktika anhand von Daten des DMKG-Kopfschmerzregisters

**DOI:** 10.1007/s00482-025-00899-1

**Published:** 2025-09-11

**Authors:** Ruth Ruscheweyh, Gudrun Goßrau, Tim Patrick Jürgens, Victoria Ruschil, Torsten Kraya, Thomas Dresler, Charly Gaul, Jörg Scheidt, Lars Neeb

**Affiliations:** 1 Kopfschmerzambulanz, Neurologische Klinik und Poliklinik, LMU München, Ziemssenstr. 1, 80336 München, Deutschland; 2 Kopfschmerzambulanz, Universitätsschmerzcentrum, Medizinische Fakultät, TU Dresden, Universitätsklinikum Dresden, Dresden, Deutschland; 3 Kopfschmerzzentrum Nordost, Neurologische Klinik und Poliklinik, Universitätsklinik Rostock, Rostock, Deutschland; 4 Neurologische Klinik, KMG Krankenhaus Güstrow, Güstrow, Deutschland; 5 Abteilung Neurologie mit Schwerpunkt Epileptologie, Universitätsklinikum Tübingen, Tübingen, Deutschland; 6 Zentrum für seltene Erkrankungen, Universitätsklinikum Tübingen, Tübingen, Deutschland; 7 Klinik für Neurologie, Klinikum St. Georg, Leipzig, Deutschland; 8 Klinik und Poliklinik für Neurologie, Universitätsklinikum Halle, Martin-Luther-Universität Halle-Wittenberg, Halle-Wittenberg, Deutschland; 9 Klinik für Psychiatrie und Psychotherapie, Universitätsklinikum Tübingen, Tübingen, Deutschland; 10 LEAD Graduiertenschule & Forschungsnetzwerk, Universität Tübingen, Tübingen, Deutschland; 11 Standort Tübingen, German Center for Mental Health (DZPG), Tübingen, Deutschland; 12 Kopfschmerzzentrum Frankfurt, Frankfurt, Deutschland; 13 Institut für Informationssysteme, Hochschule Hof, Hof, Deutschland; 14 Klinik für Neurologie, Universitätsklinikum Brandenburg an der Havel GmbH, Brandenburg an der Havel, Deutschland

**Keywords:** Migräne, CGRP(R)-Antikörper, Erstattung, Migräneprophylaxe, Vortherapie, Migraine, CGRP antibody, Insurance coverage, Migraine prevention, Prior treatments

## Abstract

**Hintergrund:**

Antikörper gegen CGRP oder seinen Rezeptor (Eptinezumab, Erenumab, Fremanezumab, Galcanezumab, im Folgenden: „CGRP(R)-Antikörper“) sind moderne, spezifische Migräneprophylaktika, die im GKV-Bereich nur für therapierefraktäre Patienten erstattungsfähig sind. Hier wurde untersucht, welchen Effekt diese Regelung auf die Auswahl der behandelten Patienten hat und wie sich die Neubewertung von Erenumab durch den Gemeinsamen Bundesausschuss ab Oktober 2022 ausgewirkt hat.

**Methode:**

Es wurden 759 Patienten mit CGRP(R)-Antikörpern mit 961 Patienten mit zugelassenen unspezifischen oralen Prophylaktika (Amitriptylin, Betablocker, Flunarizin, Topiramat) aus dem Kopfschmerzregister der Deutschen Migräne- und Kopfschmerzgesellschaft (DMKG) verglichen.

**Ergebnisse:**

Patienten mit CGRP(R)-Antikörpern hatten mehr ungenügend wirksame/verträgliche Vortherapien als Patienten mit unspezifischen oralen Prophylaktika (*p* < 0,001). Außerdem hatten sie eine längere Erkrankungsdauer (*p* < 0,001), häufiger chronische Migräne (*p* = 0,002), mehr schwere Kopfschmerztage und Akutmedikationstage (*p* = 0,004 und 0,04), geringere Lebensqualität (*p* < 0,05) sowie mehr Begleiterkrankungen (*p* = 0,001) und waren seltener berufstätig (*p* < 0,001). Nach Änderung der Erstattungsrichtlinien hat sich die Behandlung mit Erenumab hin zu weniger therapierefraktären (*p* < 0,001) Patienten mit kürzerer Erkrankungsdauer (*p* < 0,001) verschoben, die im Vergleich zu Patienten mit anderen CGRP(R)-Antikörpern weniger schwer betroffen waren (z. B. Kopfschmerztage *p* = 0,01; Beeinträchtigung *p* = 0,005).

**Schlussfolgerung:**

CGRP(R)-Antikörper werden besonders schwer betroffenen Migränepatienten verschrieben. Erstattungsvorgaben und deren Änderung haben einen deutlichen lenkenden Effekt auf den Einsatz von Migränetherapeutika.

**Zusatzmaterial online:**

Die Online-Version dieses Artikels (https://doi.org/10.1007/s00482-025-00899-1) enthält weiterführende Informationen zum Einsatz der CGRP(R)-Antikörper.

## Abstract (en)

**Background:**

Antibodies against CGRP or its receptor (eptinezumab, erenumab, fremanezumab, galcanezumab, from here on: “CGRP(R) antibodies”) are modern migraine preventives. German statutory health insurance covers CGRP(R) antibodies only for patients refractory to other preventive therapies. Thus, the effect of this regulation on patient selection was investigated, as well as the effect of a change of insurance coverage for erenumab in October 2022.

**Methods:**

In total, 759 patients with CGRP(R) antibody treatment were compared with 961 patients prescribed nonspecific oral migraine preventive medicines (amitriptyline, betablocker, flunarizine, topiramate) from the DMKG Headache Registry.

**Results:**

Patients with CGRP(R) antibodies had more prior preventive therapies that were insufficiently effective or tolerated compared to patients prescribed nonspecific oral preventive medicines (*p* < 0.001). Moreover, they had longer disease duration (*p* < 0.001), more often suffered from chronic migraine (*p* = 0.002), had more severe headache days (*p* = 0.004) and acute medication days (*p* = 0.04), lower quality of life (*p* < 0.05), more comorbidities (*p* = 0.001), and fewer of them were working (*p* < 0.001). After change of insurance coverage, treatment with erenumab shifted towards less refractory patients (*p* < 0.001) with shorter disease duration (*p* < 0.001), who were less severely affected compared to patients with other CGRP(R) antibodies (e.g., headache days *p* = 0.01; disability *p* = 0.005).

**Conclusion:**

CGRP(R) antibodies are prescribed for to patients affected by exceedingly severe migraine. Insurance coverage has a significant effect on use of medicines for migraine prevention.

## Einleitung

Seit 2018 sind in Deutschland nach und nach mehrere Antikörper gegen CGRP oder den CGRP-Rezeptor (hier zusammengefasst benannt als CGRP(R)-Antikörper) zur prophylaktischen Migränebehandlung auf den Markt gekommen (Erenumab, Fremanezumab, Galcanezumab, Eptinezumab; [1, 2]). Die CGRP(R)-Antikörper gehören zu den am besten untersuchten Migräneprophylaktika. Eine Reihe von randomisiert-kontrollierten Studien zeigt gute Wirksamkeit und meist sehr gute Verträglichkeit [3, 4]. Dies hat sich in Real-world-Studien bestätigt [5, 6].

Die CGRP(R)-Antikörper sind zugelassen für die Behandlung Erwachsener mit Migräne an mindestens 4 Tagen pro Monat. Die Kostenerstattung durch die gesetzlichen Krankenkassen wurde jedoch durch den Gemeinsamen Bundesausschuss (G-BA) beschränkt auf Patienten mit Therapieversagen oder Nichteignung von 4 (episodische Migräne) bzw. 5 (chronische Migräne) Vortherapien [7–9]. Als Versagen wird dabei eine dokumentierte unzureichende Verträglichkeit oder Wirksamkeit (nach Durchführung eines Therapieversuchs in ausreichender Dosierung und Dauer) akzeptiert. Eine Nichteignung bezieht sich z. B. auf dokumentierte Kontraindikationen. Die 4–5 Vortherapien wurden entsprechend der Zulassung für die Migräneprophylaxe festgelegt als: Amitriptylin, Betablocker, Topiramat und Flunarizin (hier zusammengefasst benannt als unspezifische orale Prophylaktika). Bei chronischer Migräne (Migräne mit ≥ 15 Kopfschmerztagen/Monat) kommt Botulinumtoxin hinzu [10]. Dadurch wurde die Behandlung mit den kostenintensiven Antikörpern auf Patienten beschränkt, für die der G‑BA anhand der vorliegenden Daten einen Zusatznutzen anerkannt hat. Für Erenumab wurde später durch eine direkte Vergleichsstudie (HER-MES) eine gegenüber Topiramat überlegene Verträglichkeit und Wirksamkeit gezeigt [11]. Daraufhin kann Erenumab nach einem erneuten G‑BA-Beschluss und nach Anerkennung einer Praxisbesonderheit durch den GKV-Spitzenverband seit Oktober 2022 bereits nach Versagen einer Vortherapie verordnet werden [12, 13]. Die Kostenerstattung im Bereich der privaten Krankenversicherungen ist nicht einheitlich geregelt, wird aber meist in Anlehnung an die Vorgaben der GKV gehandhabt.

Hier wurde anhand der Daten aus dem seit 2020 laufenden Kopfschmerzregister der Deutschen Migräne- und Kopfschmerzgesellschaft (DMKG; [14, 15]) untersucht, ob 1) infolge dieser Regelungen schwerer betroffene Patienten mit einem CGRP(R)-Antikörper behandelt werden (im Vergleich zu unspezifischen oralen Prophylaktika) und 2) ob sich dies für Erenumab nach Änderung der Erstattungssituation seit Oktober 2022 geändert hat.

## Methoden

Das DMKG-Kopfschmerzregister wird in Übereinstimmung mit der Deklaration von Helsinki durchgeführt und ist beim Deutschen Register Klinischer Studien registriert (DRKS Nr. 00021081). Das zustimmende Votum der federführenden Ethikkommission der LMU München (Nr. 20-004) sowie der für jedes teilnehmende Zentrum zuständigen Ethikkommission wurde eingeholt. Von jedem Teilnehmer wurde ein informiertes, schriftliches Einverständnis eingeholt. Das DMKG-Kopfschmerzregister schließt Kopfschmerzpatienten an teilnehmenden Praxen und Zentren in Deutschland ein und erfasst ihre Behandlung. Vor jeder Visite werden über ein Online-Portal Informationen (z. B. zu Kopfschmerzhäufigkeit, Medikation sowie einer Reihe von validierten Skalen) von Patienten eingeholt und anschließend von den Ärzten validiert bzw. ergänzt (z. B. um die Kopfschmerzdiagnose nach der Internationalen Kopfschmerzklassifikation ICHD‑3 [16]). Eine detaillierte Beschreibung findet sich bei Ruscheweyh et al. [15]. Aktuell nehmen 35 Praxen/Zentren am DMKG-Kopfschmerzregister teil.

Die hier beschriebene Auswertung erfolgte mit Datenstand vom 02.09.2024. Zu diesem Zeitpunkt hatten 3967 Patienten Daten von mindestens einer Visite im Kopfschmerzregister, davon 3672 erwachsene Patienten mit einer Migränediagnose. In die vorliegende Auswertung wurden alle erwachsenen Migränepatienten eingeschlossen, die zum Zeitpunkt einer im DMKG-Kopfschmerzregister erfassten Visite ein Prophylaktikum aus einer der folgenden 2 Gruppen verwendeten: (1) CGRP(R)-Antikörper (Eptinezumab, Erenumab, Fremanezumab, Galcanezumab) oder (2) unspezifische orale Prophylaktika (Amitriptylin, Betablocker, Topiramat, Flunarizin). Analysiert wurde jeweils die erste erfasste Visite unter dem Prophylaktikum (V1). Wo vorhanden, wurde auch die letzte Visite vor Beginn mit dem Prophylaktikum ausgewertet (V0). Diese steht nur für eine Subgruppe zur Verfügung, da manche Patienten bereits zum Zeitpunkt der 1. Visite im Kopfschmerzregister ein Prophylaktikum verwendeten. Eine stabile Komedikation mit weiteren Prophylaktika (mit Ausnahme von CGRP(R)-Antikörpern in der Gruppe mit unspezifischen Prophylaktika) war in beiden Gruppen erlaubt. Patienten, die im Verlauf der Beobachtung im Kopfschmerzregister zu unterschiedlichen Zeitpunkten einen CGRP(R)-Antikörper bzw. ein orales Prophylaktikum verwendeten, gingen in beide Gruppen ein.

Die beiden Patientengruppen (Therapie mit CGRP(R)-Antikörper vs. unspezifisches orales Prophylaktikum) wurden zum Zeitpunkt V1 bezüglich der demografischen Daten, der Migränediagnose, der Begleiterkrankungen und der Berufstätigkeit verglichen. Für Variablen, bei denen unter einer wirksamen Prophylaxe schnelle Änderungen zu erwarten sind, wurden Daten zum Zeitpunkt V0 für den Gruppenvergleich verwendet: Dies betraf die Kopfschmerzhäufigkeit, die Einnahmehäufigkeit von Schmerzmitteln, den Medikamentenübergebrauch sowie die mithilfe von validierten Skalen erhobene migränebezogene Beeinträchtigung (Migraine Disability Assessment Scale [MIDAS]; [17]), Lebensqualität (Veterans RAND 12-item Health Survey [VR-12]; [18]) und Depression, Angst und Stresssymptome (Depression, Anxiety and Stress Scales, Short Form [DASS-21]; [19]). Ein Medikamentenübergebrauch wurde entsprechend der ICHD‑3 [16] festgestellt, falls Patienten Akutschmerzmittel (inkl. Triptane) in den letzten 3 Monaten an ≥ 10 Tagen/Monat einnahmen, mit der Ausnahme alleiniger Einnahme einfacher Schmerzmittel und nichtsteroidaler Antirheumatika, für die die Grenze bei ≥ 15 Tagen/Monat lag. Eine „chronische Migräne“ bezeichnet nach ICHD‑3 eine Migräne mit ≥ 15 Kopfschmerztagen/Monat, von denen mindestens 8 die Migränecharakteristika erfüllen und/oder auf Triptane ansprechen [16].

Für die CGRP(R)-Antikörper wurden außerdem Patienten mit Beginn vor und ab Oktober 2022 verglichen sowie Patienten mit Erenumab vs. Patienten mit anderen CGRP(R)-Antikörpern.

Die statistische Auswertung wurde mit R (Version 4.0.0, https://www.r-project.org/) durchgeführt. *P* < 0,05 wurde als signifikant angesehen. Da nicht alle Variablen normalverteilt waren, wurden Gruppenvergleiche mithilfe nichtparametrischer Tests (exakter Test nach Fisher, Wilcoxon-Rangsummentest für unabhängige Stichproben) durchgeführt.

## Ergebnisse

Es wurden 759 Patienten unter Therapie mit einem CGRP(R)-Antikörper und 961 Patienten unter Therapie mit einem unspezifischen oralen Prophylaktikum eingeschlossen. Die ausgewerteten Medikamente und ggf. weitere Prophylaktika sind in Tab. 1 gezeigt. Erenumab war der am häufigsten verwendete CGRP(R)-Antikörper, Amitriptylin und Betablocker die am häufigsten verwendeten unspezifischen oralen Prophylaktika.

**Tab. 1:** Verteilung der ausgewerteten und begleitenden Prophylaktika

	CGRP(R)-Antikörper	Unspezifisches orales Prophylaktikum
*n*	759	961
*Ausgewertete Medikamente*		
–	Eptinezumab 21 (2,8 %)	Amitriptylin 355 (36,9 %)
	Erenumab 472 (62,2 %)	Betablocker 358 (37,3 %)
	Fremanezumab 178 (23,5 %)	Flunarizin 50 (5,2 %)
	Galcanezumab 99 (13,0 %)	Topiramat 251 (26,1 %)
*Kombinationstherapien*		
Patienten mit mehr als einem Prophylaktikum	183 (23,9 %)	161 (16,7 %)
(Weiteres) unspezifisches orales Prophylaktikum	103 (13,6 %)	52 (5,4 %)
Botulinumtoxin	12 (1,6 %)	24 (2,5 %)
Magnesium^$^	46 (6,0 %)	52 (5,4 %)
Andere	75 (9,9 %)	50 (5,2 %)

^$^Magnesium allein oder in Kombination mit Vitamin B2, Coenzym Q10

### Vergleich von Patienten mit CGRP(R)-Antikörpern gegenüber Patienten mit unspezifischen oralen Prophylaktika

**Abb. 1:**
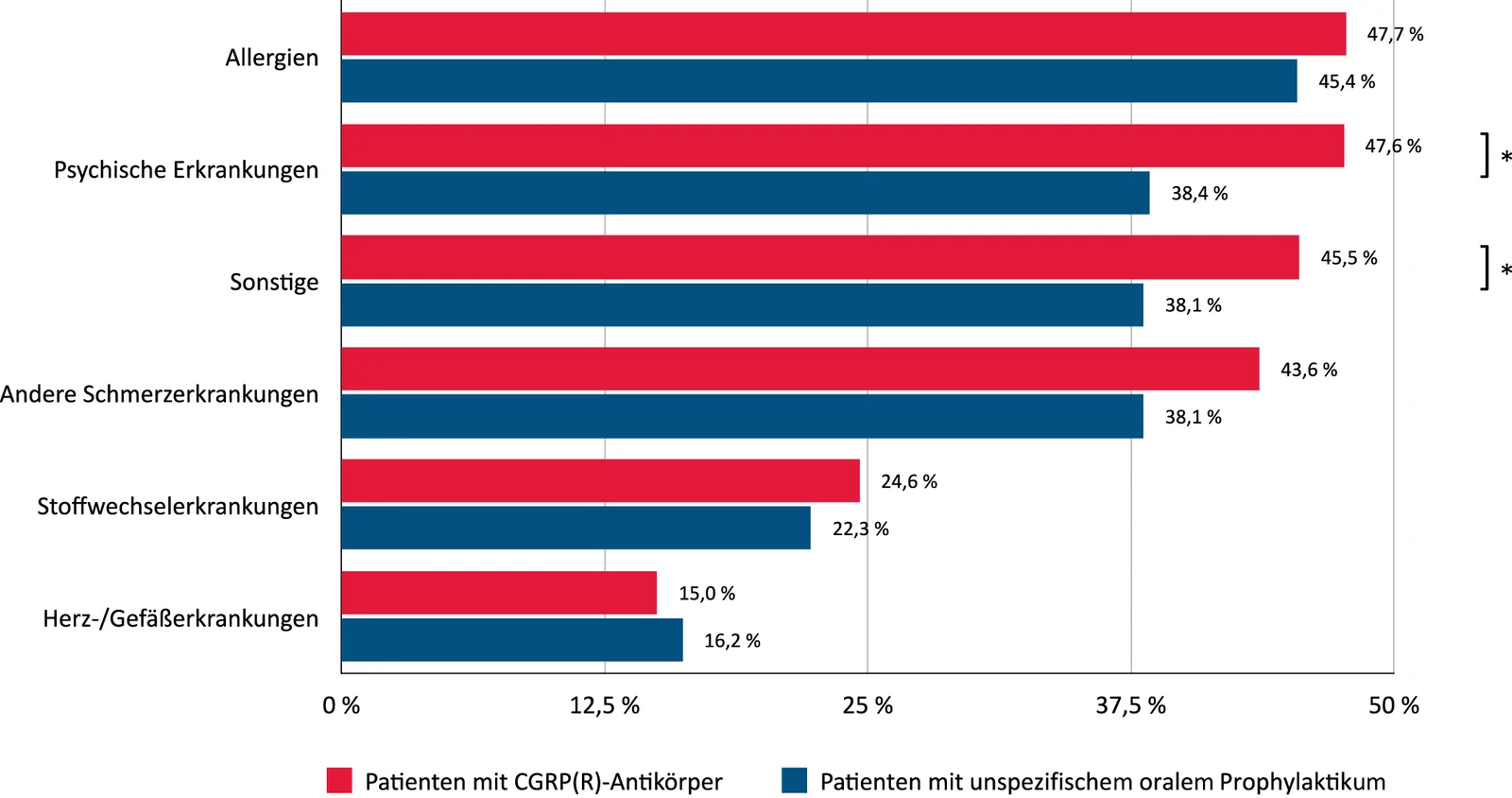
Vergleich der Begleiterkrankungen zwischen Patienten mit CGRP(R)-Antikörper und Patienten mit unspezifischem oralem Prophylaktikum. Die Sammelkategorie „Sonstige“ umfasst: Asthma bronchiale, erhöhte Nierenwerte, erhöhte Leberwerte, Gastritis, Magenblutung, Glaukom, rheumatische Erkrankungen, Epilepsie sowie Erkrankungen, die nicht in eine der erhobenen Kategorien fallen. *p < 0,05 (exakter Test nach Fisher)

**Abb. 2:**
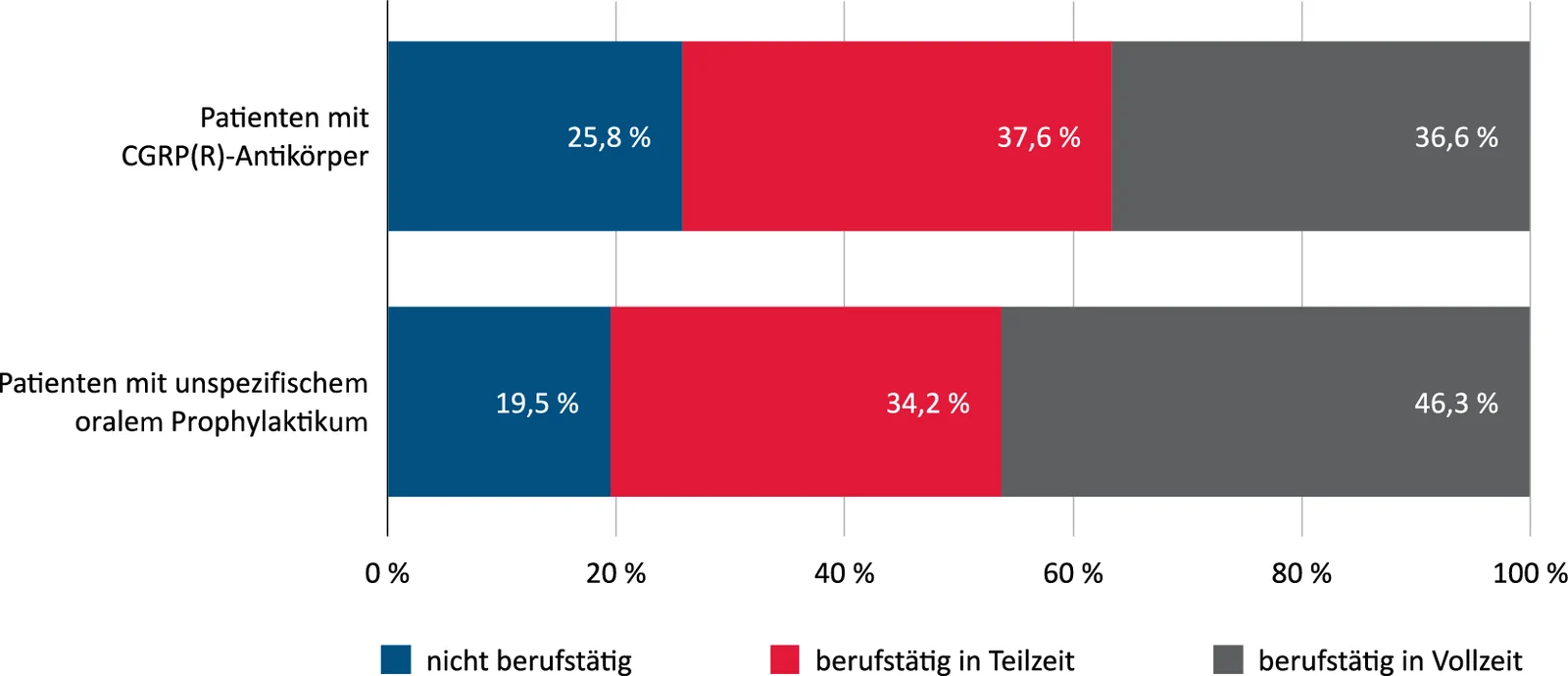
Vergleich der Berufstätigkeit zwischen Patienten mit CGRP(R)-Antikörper und Patienten mit unspezifischem oralem Prophylaktikum. *P* < 0,001 (exakter Test nach Fisher)

Die Ergebnisse des Gruppenvergleichs sind in Tab. 2 detailliert aufgeführt. Patienten mit CGRP(R)-Antikörper („CGRP-Gruppe“) waren im Schnitt 2,3 Jahre älter als Patienten mit einem unspezifischen oralen Prophylaktikum (*p* < 0,001) und hatten eine im Mittel 3,2 Jahre längere Migräneerkrankungsdauer (*p* < 0,001). In den weiteren Vergleichen zeigte sich fast durchgehend, dass Patienten aus der CGRP-Gruppe schwerer betroffen waren als Patienten der Gruppe mit unspezifischen oralen Prophylaktika. Patienten der CGRP-Gruppe hatten häufiger die Diagnose einer chronischen Migräne (+10,2 %, *p* = 0,002). Die Anzahl der Gesamtkopfschmerztage/Monat war ähnlich, aber Patienten der CGRP-Gruppe hatten mehr schwere Kopfschmerztage (im Mittel +1,4 Tage/Monat, *p* = 0,004) und mehr Tage mit Schmerzmitteleinnahme (+0,8 Tage/Monat, *p* = 0,04). Die kopfschmerzbezogene Beeinträchtigung (MIDAS-Score) war in der CGRP-Gruppe im Mittel ca. 20 % höher (*p* = 0,05). Ebenso waren Lebensqualität und subjektive Bewertung des Gesundheitszustands in der CGRP-Gruppe niedriger (alle *p* < 0,05). Symptome von Depression, Angst und Stress waren ebenfalls häufiger, dies erreichte aber keine statistische Signifikanz. Begleiterkrankungen waren signifikant häufiger in der CGRP-Gruppe (*p* < 0,001). In den einzelnen Kategorien betraf dies insbesondere die um fast 10 % häufigeren psychischen Begleiterkrankungen (*p* = 0,02; siehe Abb. 1 für weitere Kategorien). Patienten der CGRP-Gruppe waren auch seltener berufstätig, insbesondere seltener in Vollzeit tätig (−9,7 %, *p* < 0,001, Abb. 2). Wie angesichts der Erstattungsvorgaben erwartet, fand sich eine höhere Anzahl an ungenügend wirksamen oder verträglichen Vortherapien in der CGRP-Gruppe (2,3 vs. 0,7 pro Patient, *p* < 0,001). Die individuell ungeeigneten (z. B. kontraindizierten) Prophylaktika wurden hier nicht erfasst.

**Tab. 2:** Vergleich des Einsatzes von CGRP(R)-Antikörpern und unspezifischen oralen Prophylaktika bei Migränepatienten

	CGRP(R)-Antikörper	Unspezifisches orales Prophylaktikum	Statistik
*n*	759 (323^V0^)	961 (270^V0^)	–
Alter	42,7 ± 13,0	40,4 ± 12,8	***p*** **=** **0,0003**^**W**^
Weiblich/männlich/divers	88,4 %/11,2 %/0,4 %	85,7 %/14,2 %/0,1 %	*p* = 0,093^F^
*Kopfschmerzdaten*			
Migräneerkrankungsdauer (Jahre)	23,6 ± 14,0	20,4 ± 14,0	***p*** **<** **0,0001**^**W**^
Kopfschmerztage/Monat^V0^	14,8 ± 7,6	14,5 ± 7,2	*p* = 0,76^W^
Starke Kopfschmerztage/Monat^V0^	8,7 ± 5,9	7,3 ± 5,2	***p*** **=** **0,004**^**W**^
Schmerzmitteltage/Monat^V0^	8,5 ± 4,6	7,7 ± 4,5	***p*** **=** **0,04**^**W**^
Chronische Migräne	38,6 %	28,4 %	***p*** **=** **0,002**^**F**^
Medikamentenübergebrauch^V0^	33,4 %	28,1 %	*P* = 0,35^F^
Frühere Prophylaxen	1718 (2,3/Patient)	713 (0,7/Patient)	***P*** **<** **0,0001**^**W**^
*Fragebögen*			
MIDAS-Score^V0^	52,8 ± 46,4	44,4 ± 40,4	*p* = 0,05^W^
VR-12: MSC^V0^	39,5 ± 11,2	41,1 ± 11,1	***p*** **=** **0,04**^**W**^
VR-12: PSC^V0^	38,5 ± 9,5	40,6 ± 8,6	***p*** **=** **0,006**^**W**^
DASS Depression^V0^	6,4 ± 5,4	5,8 ± 5,2	*p* = 0,20^W^
DASS Angst^V0^	4,4 ± 4,5	3,9 ± 3,9	*p* = 0,46^W^
DASS Stress^V0^	8,4 ± 5,3	8,2 ± 5,2	*p* = 0,46^W^
VAS Gesundheitszustand^V0^	45,5 ± 20,8	49,5 ± 20,6	***p*** **=** **0,03**^**W**^
*Berufstätigkeit*			
Nicht/Teilzeit/Vollzeit	25,8 %/37,6 %/36,6 %	19,5 %/34,2 %/46,3 %	***p*** **=** **0,0003**^**F**^
Tage mit Arbeitsausfall/Monat^V0^	2,2 ± 3,7	2,0 ± 4,4	*p* = 0,11^W^
*Begleiterkrankungen*			
Anzahl angegebene Begleiterkrankungen pro Patient	4,5 ± 4,3	3,8 ± 3,6	***P*** **=** **0,0007**^**W**^
Weitere Schmerzerkrankungen	331 (43,6 %)	366 (38,1 %)	*p* = 0,14^F^
Psychische/psychiatrische Erkrankungen	361 (47,6 %)	369 (38,4 %)	***p*** **=** **0,02**^**F**^

Ausgewertet wurde die erste dokumentierte Visite unter dem ausgewerteten Prophylaktikum (V1), außer bei den mit V0 gekennzeichneten Werten, dort wurde die letzte Visite vor Beginn mit dem ausgewerteten Prophylaktikum herangezogen. Signifikante Ergebnisse sind fett gedruckt

*MIDAS* Migraine Disability Assessment (migränebezogene Beeinträchtigung); *VR-12* Veterans RAND 12 (Lebensqualität); *MSC* mentale Summenskala; *PSC* physische Summenskala, *DASS* Depression-Angst-Stress-Skalen, *VAS* visuelle Analogskala

^W^„Wilcoxon rank sum test“ für unabhängige Stichproben

^F^„Fisher exact test“

Zusammenfassend waren Patienten mit einem CGRP(R)-Antikörper schwerer von ihrer Migräne betroffen, hatten mehr Begleiterkrankungen (v. a. psychische Erkrankungen) und waren seltener in Vollzeit berufstätig.

### Vergleich von Patienten mit Erenumab vor und ab Oktober 2022 (Änderung der Erstattungsfähigkeit)

Im nächsten Schritt wurden mit Erenumab behandelte Patienten vor (*n* = 173) und nach (*n* = 298) Änderung der Erstattungssituation untersucht (Tabelle S1 im Online-Zusatzmaterial). Die Anzahl der gescheiterten Vortherapien sank erwartungsgemäß ab, von 2,6 auf 1,6 pro Patient (*p* < 0,001), ebenso die Erkrankungsdauer (im Mittel 7,3 Jahre kürzer, *p* < 0,001), dagegen stieg die Anzahl der Berufstätigen an (in Vollzeit Berufstätige +23,5 %, *p* < 0,001). Darüber hinaus fand sich ab Oktober 2022 in vielen Parametern eine Tendenz hin zu weniger stark betroffenen Patienten (z. B. MIDAS-Score −10,3 Punkte, *p* = 0,06; starke Kopfschmerztage −1,4 Tage/Monat, *p* = 0,17; Begleiterkrankungen −0,6 pro Patient, *p* = 0,15), auch wenn dies aufgrund der kleineren Fallzahlen keine statistische Signifikanz erreichte (Tabelle S1 im Online-Zusatzmaterial).

Zusammenfassend fand sich nach Änderung der Erstattungssituation für Erenumab eine Verschiebung hin zu weniger therapierefraktären, weniger lang von der Migräne betroffenen und öfter berufstätigen Patienten. Weitere Parameter wiesen auf eine (statistisch nicht signifikante) Verschiebung zu weniger schwer betroffenen Patienten hin.

### Vergleich von Patienten mit anderen CGRP(R)-Antikörpern vor und nach Oktober 2022

Für die anderen CGRP(R)-Antikörper (Eptinezumab, Fremanezumab, Galcanezumab) gab es keine statistisch signifikanten Gruppenunterschiede von vor zu nach Oktober 2022 (vor: *n* = 205, nach: *n* = 90; Tabelle S2 im Online-Zusatzmaterial). Nominal zeigt sich eine leichte (im Vergleich zu Erenumab aber weniger ausgeprägte) Abnahme der Migränedauer (−1,9 Jahre, n. s.) sowie eine Verschiebung hin zu mehr Berufstätigkeit (+10 % mehr in Vollzeit berufstätig).

### Vergleich Patienten mit Erenumab gegenüber Patienten mit anderen CGRP(R)-Antikörpern ab Oktober 2022 (Änderung der Erstattungsfähigkeit)

Der direkte Vergleich im Zeitraum ab Oktober 2022 ergab, dass mit Erenumab behandelte Patienten (*n* = 298) im Mittel signifikant weniger schwer betroffen waren als Patienten mit Eptinezumab, Fremanezumab oder Galcanezumab (*n* = 90, Tabelle S3 im Online-Zusatzmaterial). Das betraf z. B. die Kopfschmerztage im Monat (−2,9 Tage, *p* = 0,01), den MIDAS-Score (−17,2 Punkte, *p* = 0,005), die Anzahl der Begleiterkrankungen (−1,4/Patient, *p* = 0,009) sowie die Berufstätigkeit (+13,8 % Berufstätige, +9,2 % in Vollzeit, *p* = 0,01). Es zeigte sich außerdem, dass die im DMKG-Kopfschmerzregister erfasste Verwendung von Erenumab gegenüber der von Eptinezumab, Fremanezumab und Galcanezumab ab Oktober 2022 deutlich angestiegen ist. Vor Oktober 2022 waren 45,8 % der CGRP(R)-Antikörper-Patienten mit Erenumab behandelt, im Zeitraum danach stieg dieser Prozentsatz auf 76,8 %.

Zusammenfassend wurde Erenumab nach Änderung der Erstattungssituation im Mittel bei signifikant weniger schwer betroffenen Patienten verwendet als Eptinezumab, Fremanezumab und Galcanezumab.

## Diskussion

Die wichtigsten Ergebnisse der Studie sind:

- Migränepatienten, die einen CGRP(R)-Antikörper erhalten, sind signifikant schwerer betroffen als solche mit einem unspezifischen oralen Prophylaktikum.
- Mit Änderung der Erstattungssituation ab Oktober 2022 gab es eine signifikante Änderung der Anwendung von Erenumab hin zu weniger therapierefraktären und weniger schwer betroffenen Patienten.

Diese Ergebnisse zeigen, dass Patienten, die einen CGRP(R)-Antikörper erhalten, nicht nur eine größere Anzahl gescheiterter Vortherapien haben. Tatsächlich sind sie auch anhand zahlreicher anderer klinischer Parameter signifikant schwerer betroffen als Patienten, die ein unspezifisches orales Prophylaktikum erhalten. Dies zeigt im Ergebnis nicht nur einen wirtschaftlichen, sondern auch einen zielgerichteten, medizinisch begründeten Einsatz dieser im oberen Preissegment angesiedelten Medikamente.

Bemerkenswert ist außerdem, dass sich der Gruppenunterschied nicht nur an den unmittelbaren Kopfschmerzparametern wie der Anzahl der starken Kopfschmerztage und Schmerzmitteltage/Monat zeigt, sondern sich durch alle Domänen inkl. Beeinträchtigung, Lebensqualität, Begleiterkrankungen und Berufstätigkeit zieht. Dies belegt nochmals, dass Migräne Auswirkungen auf sämtliche Lebensbereiche hat. Bezüglich der Begleiterkrankungen sind zahlreiche Komorbiditäten der Migräne bekannt [20, 21]. Die hier gezeigten Daten weisen aber erneut darauf hin, dass die Schwere einer Migräne besonders eng mit der psychischen Komorbidität zusammenhängt [22, 23].

Bemerkenswert an den oben gezeigten Daten ist auch, dass Patienten mit Versagen mehrerer Vortherapien im Mittel schwerer von Migräne betroffen sind. Es könnte sein, dass schwer betroffene Patienten per se ein schlechteres Ansprechen auf (unterschiedliche) Migräneprophylaktika zeigen, das im Verlauf konstant bleibt. Andererseits wird oft diskutiert, ob Patienten mit bereits hoher Kopfschmerzfrequenz ohne wirksame Migräneprophylaxe über die Zeit zur Progredienz neigen, sodass die Durchführung mehrerer unwirksamer Therapieversuche schließlich in einer Verschlechterung resultieren kann. In diesem Fall wäre es ethisch bedenklich, diesen Patienten eine wirksame und gut untersuchte, spezifische Therapie bis zum Abschluss der Therapieversuche mit unspezifischen, deutlich weniger gut untersuchten und nebenwirkungsreicheren Pharmaka vorzuenthalten. Hier sollte die effektive Wirtschaftlichkeit prospektiv untersucht werden vor dem Hintergrund eines erhöhten Chronifizierungsrisikos bei erfolglosen Therapien. In der Tat hat eine multizentrische europäische Real-world-Studie mit > 5800 Patienten gezeigt, dass weniger schwer betroffene, weniger therapierefraktäre Patienten besser auf CGRP(R)-Antikörper ansprechen [6]. Ein schlechteres Ansprechen auf CGRP(R)-Antikörper bei Patienten mit Versagen vieler Vortherapien ist auch in mehreren kleineren Real-world-Studien berichtet worden [24–26]. Tatsächlich hat eine Studie der Charité zeigen können, dass der Effekt von Erenumab nach Änderung der Erstattungsrichtlinien (und konsekutiv Behandlung von Patienten mit weniger gescheiterten Vortherapien) signifikant besser war als vorher [27]. Allerdings sind früh und spät behandelte Patientenkollektive in dieser Studie nicht direkt vergleichbar, da letztere Gruppe nur therapierefraktäre Patienten enthält.

Unsere Daten zeigen auch, dass sich seit Anerkennung der Praxisbesonderheit im Oktober 2022 die Anwendung von Erenumab hin zu weniger therapierefraktären, weniger schwer betroffenen Patienten mit weniger langer Migränevorgeschichte verschoben hat. Bei den anderen CGRP(R)-Antikörpern ist dagegen keine signifikante Verschiebung des Einsatzes zu beobachten. Daher ist davon auszugehen, dass die Verschiebung bei Erenumab Folge der geänderten Erstattungssituation ist und nicht eines generell früheren Einsatzes von CGRP(R)-Antikörpern. Wenig überraschend zeigt sich auch, dass seit Oktober 2022 die Verwendung von Erenumab gegenüber den anderen CGRP(R)-Antikörpern deutlich zugenommen hat, da Erenumab früher eingesetzt werden kann. Auch wenn dies die bessere Evidenzlage für Erenumab widerspiegelt, gibt es klinisch wenig Unterschiede zwischen den verschiedenen CGRP(R)-Antikörpern [28, 29].

### Limitationen

Zu berücksichtigen ist, dass am DMKG-Kopfschmerzregister vorwiegend Kopfschmerzzentren und Praxen mit besonderem Kopfschmerzinteresse teilnehmen, sodass die eingeschlossenen Patienten im Vergleich zu Migränepatienten aus der Allgemeinbevölkerung überdurchschnittlich schwer betroffen waren (zu sehen z. B. an der mittleren Kopfschmerzhäufigkeit von fast 15 Tagen/Monat), auf Behandlerseite aber auch eine höhere Bereitschaft und mehr Erfahrung mit der Verordnung von CGRP(R)-Antikörpern bestand. Andererseits konnten dadurch überhaupt erst ausreichend Patienten mit CGRP(R)-Antikörper-Behandlung für die hier durchgeführte Auswertung eingeschlossen werden. Auch wurde in der vorliegenden Arbeit nicht untersucht, ob die Änderung der Erstattungspolitik zu einem besseren Outcome bezüglich der Kopfschmerzen geführt hat. Dies wird in einer separaten Arbeit erfolgen.

## Fazit für die Praxis

- Daten aus dem DMKG-Kopfschmerzregister haben gezeigt, dass Patienten, die einen CGRP(R)-Antikörper erhalten, im Mittel signifikant schwerer betroffen sind als Patienten, die eine unspezifische orale Migräneprophylaxe erhalten.
- Dies hat sich nach der Änderung der Erstattungsfähigkeit von Erenumab seit Oktober 2022 hin zu etwas weniger therapierefraktären, weniger schwer betroffenen Patienten mit kürzerer Migräneerkrankung verschoben.
- Insgesamt zeigen die Daten, dass Erstattungsvorgaben und deren Änderungen einen schnellen und signifikanten Effekt auf die Verwendung von Migränetherapien haben können.

## Supplementary Information

Weiterführende Information zum Einsatz der CGRP(R)-Antikörper

## Data Availability

Die Datenhoheit für die Daten aus dem Kopfschmerzregister liegt bei der DMKG. Auf begründete, wissenschaftliche Anfrage hin wird Einsicht in die Datenauswertung gewährt bzw. die verwendeten Daten zur Verfügung gestellt.
