# Supplementary material for: Migraine patients treated with CGRP(R) antibodies: Are they different from patients treated with nonspecific oral prophylaxis? Analysis from the DMKG headache registry
Source: Schmerz. 2025 Sep 11;40(5):369–76. [Article in German] doi: 10.1007/s00482-025-00899-1 (PMC13597623; doi:10.1007/s00482-025-00899-1)
Supplement: Supplementary file 1 [file 482_2025_899_MOESM1_ESM.pdf]

Tabelle S1: Vergleich des Einsatzes von Erenumab vor und nach der Änderung der Erstattungsfähigkeit

	**Vor Oktober 2022**	**Ab Oktober 2022**	**Statistik**
**n**	173 (45[V0])	298 (158[V0])	
Alter	46,0 ± 13,1	39,1 ± 11,5	**p < 0,0001[W]**
Weiblich / männlich / divers	86,1% / 13,3% / 0,6%	88,6% / 11,1% /0,3%	p = 0,57[F]
**Kopfschmerzdaten**			
Migräne-Erkrankungsdauer (in Jahren)	27,4 ± 14,5	20,1 ± 12,8	**p < 0,0001[W]**
Kopfschmerztage/Monat[V0]	14,5 ± 7,5	14,2 ± 7,8	p = 0,58[W]
Starke Kopfschmerztage/Monat[V0]	9,4 ± 6,5	8,0 ± 5,6	p = 0,17[W]
Schmerzmitteltage/Monat[V0]	8,3 ± 4,7	8,3 ± 4,7	p = 0,96[W]
Chronische Migräne	70 (40,5%)	102 (34,2%)	P = 0,36[F]
Medikamentenübergebrauch[V0]	28,9%	30,4%	p = 1,00[F]
Frühere Prophylaxen	458 (2,6/Patient)	491 (1,6/Patient)	**p = 0,0001[W]**
**Fragebögen**			
MIDAS-Score[V0]	57,4 ± 45,8	47,1 ± 46,7	p = 0,06[W]
VR-12: MSC[V0]	39,3 ± 9,7	40,2 ± 11,5	p = 0,53[W]
VR-12: PSC[V0]	38,7 ± 9,8	39,8 ± 8,9	p = 0,60[W]
DASS Depression[V0]	6,5 ± 5,2	6,2 ± 5,6	p = 0,32[W]
DASS Angst[V0]	4,2 ± 4,6	4,3 ± 4,3	p = 0,88[W]
DASS Stress[V0]	8,6 ± 5,2	8,0 ± 5,1	p = 0,56[W]
VAS Gesundheitszustand[V0]	44,4 ± 20,4	47,6 ± 22,2	p = 0,38[W]
**Berufstätigkeit**			
Keine / Teilzeit / Vollzeit	32,4% / 34,1% / 33,5%	15,1% / 37,9% / 47,0%	**p < 0,0001[F]**
Tage mit Arbeitsausfall/Monat[V0]	1,9 ± 2,8	2,0 ± 3,6	p = 0,87[W]
**Begleiterkrankungen**			
Anzahl Begleiterkrankungen	4,5 ± 4,2	3,9 ± 3,9	p = 0,15[W]
Andere Schmerzerkrankungen	76 (43,9%)	114 (38,3%)	p = 0,47[F]
Psychische/psychiatrische Erkrankungen	86 (49,7%)	120 (40,3%)	p = 0,23[F]

Ab Oktober 2022 wurde die Erstattungsfähigkeit von Erenumab geändert, statt 4-5 gescheiterter Vortherapien ist seitdem nur noch 1 gescheiterte Vortherapie gefordert. Ausgewertet wurde die erste dokumentierte Visite unter dem ausgewerteten Prophylaktikum (V1), außer bei den mit V0 gekennzeichneten Werten, dort wurde die letzte Visite vor Beginn mit dem ausgewerteten Prophylaktikum herangezogen. [W]Wilcoxon rank sum test für unabhängige Stichproben. [F]Fisher Exact Test. Signifikante Ergebnisse sind fett gedruckt. MIDAS, Migraine Disability Assessment (migränebezogene Beeinträchtigung); VR-12, Veterans Rand 12 (Lebensqualität); MSC Mentale Summenskala; PSC Physische Summenskala, DASS, Depression Angst Stress Skalen, VAS, Visuelle Analogskala.

Tabelle S2: Vergleich des Einsatzes von Fremanezumab, Galcanezumab und Eptinezumab vor und nach Oktober 2022

	**Vor Oktober 2022**	**Ab Oktober 2022**	**Statistik**
**n**	205 (80^{V0})	90 (45^{V0})	
Alter	45,1 ± 13,2	43,1 ± 13,5	$p = 0{,}23^{W}$
Weiblich / männlich / divers	90,2% / 9,3% / 0,5%	88,9% / 11,1% / 0,0%	$p = 0{,}77^{F}$
**Kopfschmerzdaten**			
Migräne-Erkrankungsdauer (in Jahren)	25,4 ± 14,2	23,5 ± 15,5	$p = 0{,}14^{W}$
Kopfschmerztage/Monat^{V0}	14,8 ± 7,8	17,1 ± 7,5	$p = 0{,}08^{W}$
Starke Kopfschmerztage/Monat^{V0}	8,5 ± 5,4	10,3 ± 6,6	$p = 0{,}14^{W}$
Schmerzmitteltage/Monat^{V0}	8,4 ± 4,3	9,1 ± 4,9	$p = 0{,}57^{W}$
Chronische Migräne	79 (38,5%)	46 (51,1%)	$p = 0{,}21^{F}$
Medikamentenübergebrauch^{V0}	38,8%	35,6%	$p = 0{,}86^{F}$
Frühere Prophylaxen	555 (2,7/Patient)	227 (2,5/Patient)	$p = 0{,}47^{W}$
**Fragebögen**			
MIDAS-Score^{V0}	55,4 ± 43,4	64,3 ± 47,6	$p = 0{,}32^{W}$
VR-12: MSC^{V0}	39,6 ± 11,9	37,3 ± 9,7	$p = 0{,}29^{W}$
VR-12: PSC^{V0}	37,9 ± 10,5	34,9 ± 8,6	$p = 0{,}09^{W}$
DASS Depression^{V0}	6,3 ± 5,5	7,2 ± 5,0	$p = 0{,}19^{W}$
DASS Angst^{V0}	4,6 ± 4,7	4,8 ± 5,2	$p = 0{,}80^{W}$
DASS Stress^{V0}	9,0 ± 5,5	9,4 ± 5,7	$p = 0{,}70^{W}$
VAS Gesundheitszustand^{V0}	44,9 ± 21,9	39,2 ± 18,0	$p = 0{,}17^{W}$
**Berufstätigkeit**			
Keine / Teilzeit / Vollzeit	34,6% / 37,6% / 27,8%	28,9% / 33,3% / 37,8%	$p = 0{,}24^{F}$
Tage mit Arbeitsausfall/Monat^{V0}	2,4 ± 4,4	2,6 ± 3,0	$p = 0{,}32^{W}$
**Begleiterkrankungen**			
Anzahl angegebene Begleiterkrankungen pro Patient	5,1 ± 4,6	5,3 ± 4,5	$p = 0{,}68^{W}$
Weitere Schmerzerkrankungen	96 (46,8%)	48 (53,3%)	$p = 0{,}58^{F}$
Psychische/psychiatrische Erkrankungen	106 (51,7%)	51 (56,6%)	$p = 0{,}67^{F}$

Ausgewertet wurde die erste dokumentierte Visite unter dem ausgewerteten Prophylaktikum (V1), außer bei den mit V0 gekennzeichneten Werten, dort wurde die letzte Visite vor Beginn mit dem ausgewerteten Prophylaktikum herangezogen. ^{W}Wilcoxon rank sum test für unabhängige Stichproben. ^{F}Fisher Exact Test. Signifikante Ergebnisse sind fett gedruckt. MIDAS, Migraine Disability Assessment (migränebezogene Beeinträchtigung); VR-12, Veterans Rand 12 (Lebensqualität); MSC Mentale Summenskala; PSC Physische Summenskala, DASS, Depression Angst Stress Skalen, VAS, Visuelle Analogskala.

Tabelle S3: Vergleich des Einsatzes von Erenumab mit dem Einsatz der anderen CGRP-Antikörper (Fremanezumab, Galcanezumab und Eptinezumab) ab Oktober 2022

	**Erenumab Ab Oktober 2022**	**Andere Ak Ab Oktober 2022**	**Statistik**
**n**	298 (158^{V0})	90 (45^{V0})	
Alter	39,1 ± 11,5	43,1 ± 13,5	**p = 0,01^{W}**
Weiblich / männlich / divers	88,6% / 11,1% / 0,3%	88,9% / 11,1% / 0,0%	p = 1,00^{F}
**Kopfschmerzdaten**			
Dauer der Migräne (in Jahren)	20,1 ± 12,8	23,5 ± 15,5	p = 0,16^{W}
Kopfschmerztage/Monat^{V0}	14,2 ± 7,8	17,1 ± 7,5	**p = 0,01^{W}**
Starke Kopfschmerztage/Monat^{V0}	8,0 ± 5,6	10,3 ± 6,6	**p = 0,02^{W}**
Schmerzmitteltage/Monat^{V0}	8,3 ± 4,7	9,1 ± 4,9	p = 0,29^{W}
Chronische Migräne	102 (34,2%)	46 (51,1%)	P = 0,08^{F}
Medikamentenübergebrauch^{V0}	30,4%	35,6%	P = 0,61^{F}
Frühere Prophylaxen	491 (1,6/Patient)	227 (2,5/Patient)	**p = 0,02^{W}**
**Fragebögen**			
MIDAS-Score^{V0}	47,1 ± 46,7	64,3 ± 47,6	**p = 0,005^{W}**
VR-12: MSC^{V0}	40,2 ± 11,5	37,3 ± 9,7	p = 0,11^{W}
VR-12: PSC^{V0}	39,8 ± 8,9	34,9 ± 8,6	**p = 0,0007^{W}**
DASS Depression^{V0}	6,2 ± 5,6	7,2 ± 5,0	p = 0,08^{W}
DASS Angst^{V0}	4,3 ± 4,3	4,8 ± 5,2	p = 0,57^{W}
DASS Stress^{V0}	8,0 ± 5,1	9,4 ± 5,7	p = 0,19^{W}
VAS Gesundheitszustand^{V0}	47,6 ± 22,2	39,2 ± 18,0	**p = 0,02^{W}**
**Berufstätigkeit**			
Keine / Teilzeit / Vollzeit	15,1% / 37,9% / 47,0%	28,9% / 33,3% / 37,8%	**p = 0,01^{F}**
Tage mit Arbeitsausfall/Monat^{V0}	2,0 ± 3,6	2,6 ± 3,0	p = 0,27^{W}
**Begleiterkrankungen**			
Anzahl angegebene Begleiterkrankungen pro Patient	3,9 ± 3,9	5,3 ± 4,5	**p = 0,009^{W}**
Weitere Schmerzerkrankungen	114 (38,3%)	48 (53,3%)	p = 0,13^{F}
Psychische/psychiatrische Erkrankungen	120 (40,3%)	51 (56,6%)	p = 0,11^{F}

Ausgewertet wurde die erste dokumentierte Visite unter dem ausgewerteten Prophylaktikum (V1), außer bei den mit V0 gekennzeichneten Werten, dort wurde die letzte Visite vor Beginn mit dem ausgewerteten Prophylaktikum herangezogen. ^{W}Wilcoxon rank sum test für unabhängige Stichproben. ^{F}Fisher Exact Test. Signifikante Ergebnisse sind fett gedruckt. MIDAS, Migraine Disability Assessment (migränebezogene Beeinträchtigung); VR-12, Veterans Rand 12 (Lebensqualität); MSC Mentale Summenskala; PSC Physische Summenskala, DASS, Depression Angst Stress Skalen, VAS, Visuelle Analogskala.
